# Functional hyperthermia and comorbid psychiatric disorders

**DOI:** 10.1186/s13030-023-00295-0

**Published:** 2023-11-13

**Authors:** Takakazu Oka

**Affiliations:** Department of Psychosomatic Medicine, International University of Health and Welfare Narita Hospital, 852 Hatakeda, Narita, 286-8520 Chiba Japan

## Commentary letter

In the latest issue of the journal Biopsychosocial Medicine, the commentary by Dr. Ginier-Gillet provides a historical overview of functional hyperthermia (FH) [1]. He describes how FH, i.e., hyperthermia without inflammation, was recognized when neither the mechanisms of infection-induced fever and stress-induced hyperthermia [2] nor the effects of acute and chronic stress on body temperature [3, 4] were fully understood. He reports that FH was classified as a neurosis in the 1920s and 30s, even though low-grade fever/hyperthermia was a physical symptom. Reimann divided patients with low-grade fever into two groups: those with habitual hyperthermia, which is analogous to FH; and those with neurosis. He found that patients with neurosis showed a higher burden of symptoms, whereas those with habitual hyperthermia showed active coping. His observations can now be revised as follows: Patients with FH are divided into those with and without comorbid psychiatric disorders. Patients with comorbid psychiatric disorders correspond to patients with neurosis. These patients have a higher burden of symptoms, whereas patients without such comorbidities display more active coping.

Psychiatric disorders may not only affect the coping patterns of patients with FH, but also, by themselves, affect both core body temperature and the severity of the stress-induced hyperthermic response. Both effects may lead to the development of FH. For example, core body temperature, measured rectally or vaginally, has been found to be higher in depressed study patients than in healthy study controls, especially during sleep. The depression score was positively correlated with the 24-hour core body temperature [5]. There have been case reports in which the authors surmised that patients’ anxiety over or fear of contracting COVID-19 [6] or transmitting it to others [7] may have led to FH. One report described a patient with bipolar II disorder who developed a marked increase in axillary temperatures from 37.6 to 39.3 °C during a stressful 45-min interview, although the changes in pyrogenic cytokine levels were minimal [8].

Therefore, with the caveat that the Diagnostic and Statistical Manual of Mental Disorders (DSM-5) be used to enroll patients with the proper classification instead of neurosis, it would be interesting to conduct a study on the prevalence of comorbid psychiatric disorders in patients with FH. Okada et al. have already reported that neurodevelopmental disorders are observed in 25% of pediatric patients with FH [9, 10]. Recent studies have also demonstrated that, in addition to psychiatric disorders, orthostatic dysregulation – especially in subtypes of patients with instantaneous orthostatic hypotension [11] and postural orthostatic tachycardia syndrome [12–14] – is common in patients with FH. These comorbid diseases may share the common pathophysiological mechanisms involved in FH or affect the prognosis of patients with FH, as well as their symptom burdens and coping patterns.

We must also take comorbid psychiatric disorders into account when treating patients with FH. Since multiple factors are involved in the development of FH, patients with FH should be treated on the basis of multidimensional perspectives. For patients with psychogenic fever (FH associated with acute or chronic psychosocial stress), therapeutic strategies include the following: disease education; lifestyle guidance encompassing the adjustment of their circadian rhythm, improvement of their stress management skills, and changes to their environment; psychotherapy; psychophysiological techniques to elicit a relaxation response; pharmacotherapy; and treatment of comorbid physical and psychiatric diseases [15].

Most patients with adolescent psychogenic fever but without psychiatric disease are “over-adapted” children who are obedient, well-behaved, and high-achieving. Therefore, their treatment includes reducing feelings of guilt about not being perfect or needing to take breaks, reducing their level of effort from 100 to 70%, and encouraging self-assertion. In contrast, for the treatment of patients with neurodevelopmental disorders who have psychogenic fever, it is important to set specific activity goals, as it is difficult for such patients to determine what is appropriate and to adjust their environment to suit their specific needs [9, 10].

Thus, adopting a biopsychosocial perspective is essential to understanding the　pathophysiological mechanisms underlying FH and developing corresponding treatment plans.

## Data Availability

Data sharing is not applicable.

## Declarations

### Trial registration

The study was not registered as a trial.
